# Supplementary material for: Antireflective vertical-cavity surface-emitting laser for LiDAR
Source: Nat Commun. 2024 Feb 6;15:1105. doi: 10.1038/s41467-024-44754-w (PMC10847414; doi:10.1038/s41467-024-44754-w)
Supplement: Supplementary file 1 — Supplementary Information [file 41467_2024_44754_MOESM1_ESM.pdf]

Supplementary Information of

"Antireflective vertical-cavity surface-emitting laser for LiDAR"

Cheng Zhang[1], Huijie Li[1], Dong Liang[1]*

[1]Vertilite Co. Ltd, Wujin District, Changzhou, Jiangsu, China

*Email: ld@vertilite.com

A. 6J 250 µm AR-VCSEL epi structure, device layout

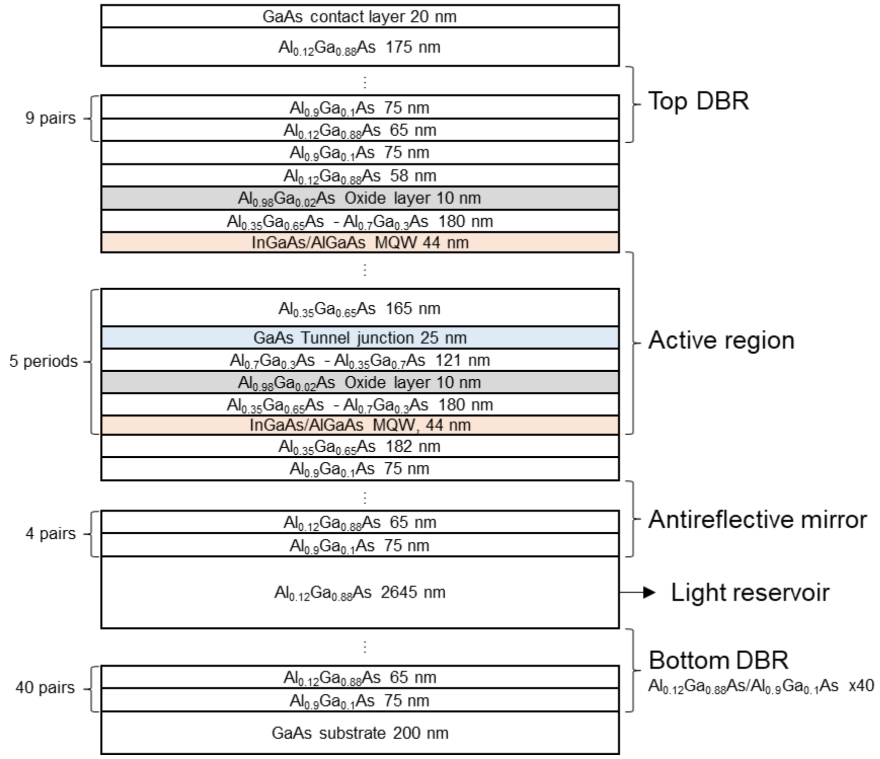

Figure S1 | A detailed epitaxial structure of the 6J AR-VCSEL in Figure 2a in the main text. Compositional graded layers between DBR layers are omitted for clarity purpose.

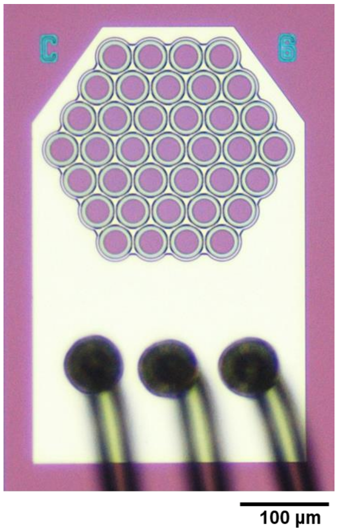

Figure S2 | A fully fabricated device image of the AR-VCSEL in Figure 2a.

B. Constructing the electric field distribution in an AR-VCSEL

In the following diagrams, we prepared a series of conceptual VCSEL designs to facilitate the understanding of how the electric field is established in an AR-VCSEL.

Firstly, in Figure S3(a), a simple 0.5λ cavity VCSEL with an N- and a P-DBR is illustrated with the standing wave electric field intensity and refractive index profiles. Both the N- and P-DBR consist of ¼ λ layers with alternating refractive indices. The relative E-field intensity inside the cavity (>50) is very high compared to E-field at the two ends of the structure, where it is set to be Unity.

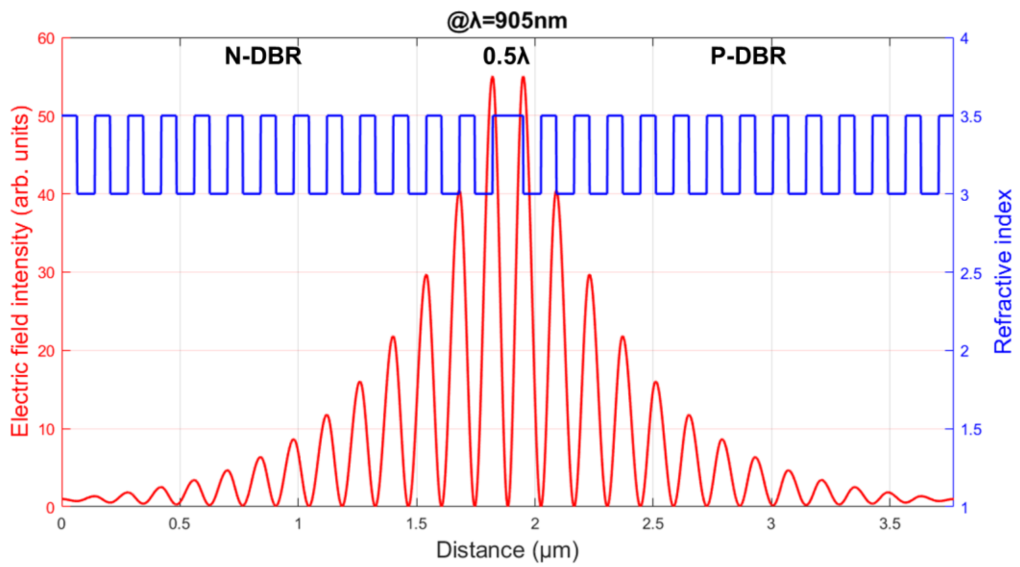

Figure S3(a). Standard 0.5λ cavity. The number of P- and N- DBR pairs are set the same for simplicity.

Secondly, in Figure S3(b), we extend the cavity length from 0.5λ to 10.5λ. Any cavity length of (1/2 + m/2) λ (m is a positive integer) does not disturb the E-field distribution in the DBRs.

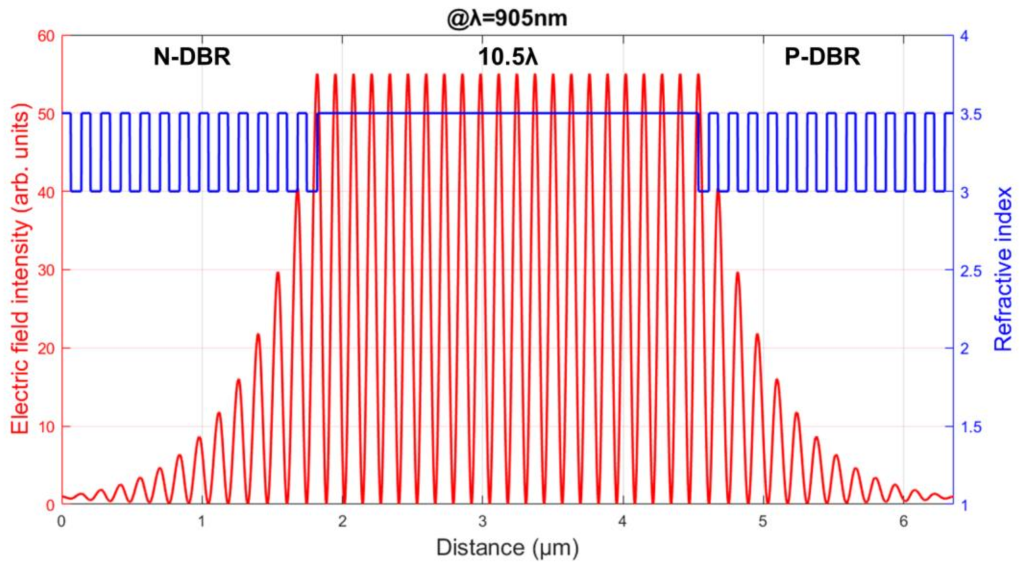

Figure S3(b). A standard 10.5λ cavity.

Thirdly, in Figure S3(c), we expand the thickness of one of the P-DBR layers from ¼ λ to ¾ λ. Note that this spacer layer thickness can be (¼ + m/2) λ, where m is a positive integer, without affecting the electric field intensity in anywhere else. Adding (m/2) λ inside a DBR structure does not create another resonant cavity.[1,2]

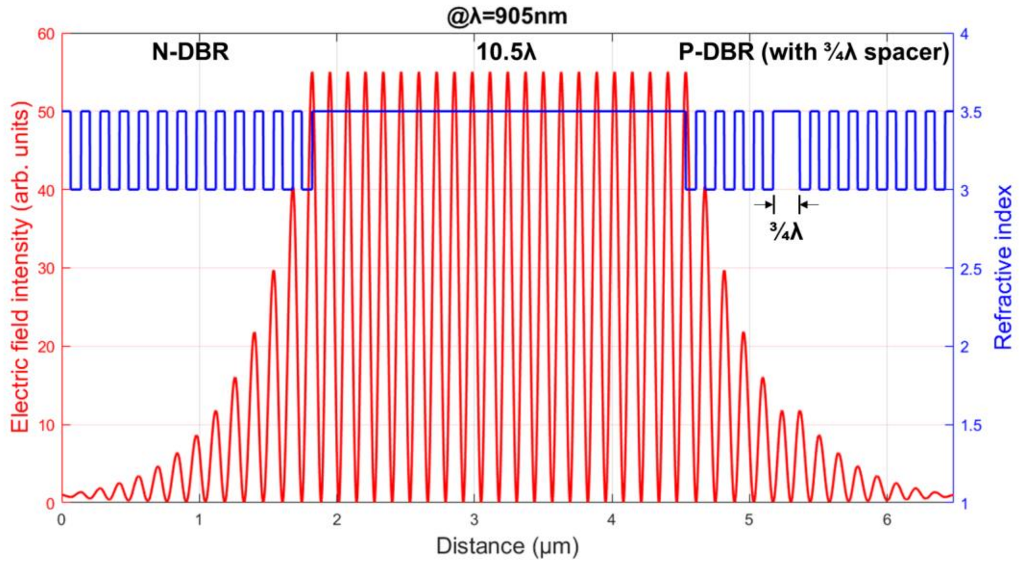

Figure S3(c). A 10.5λ cavity with a ¾ λ spacer inside P-DBR.

Then in Figure S3(d) we expand the ¾ λ layer's thickness in the P-DBR to 11¾ λ. The electric field intensity profile remains the same in anywhere else.[1,2]

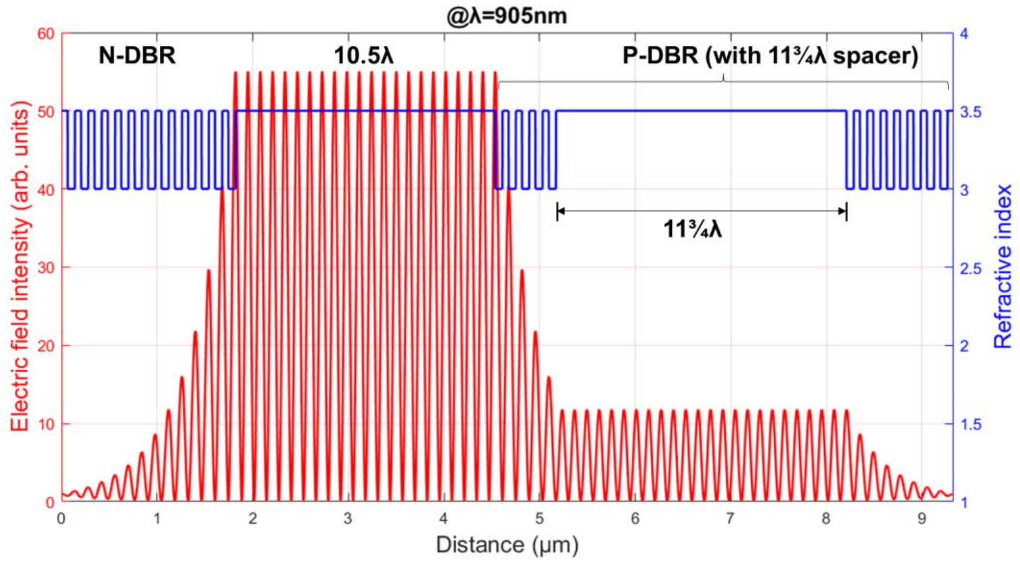

Figure S3(d). A 10.5λ cavity with a 11¾ λ spacer inside P-DBR.

Lastly in Figure S3(e), we replace the 11¾ λ spacer layer with the active region, which has the same optical thickness of 11¾ λ. By carefully placing the multi-quantum wells in the antinodes of the E-field standing waves, while tunnel junctions and oxide layers in the nodes, the electric field distribution can be mostly maintained. No additional cavity is added in this process. The original 10.5λ cavity is now the light reservoir.

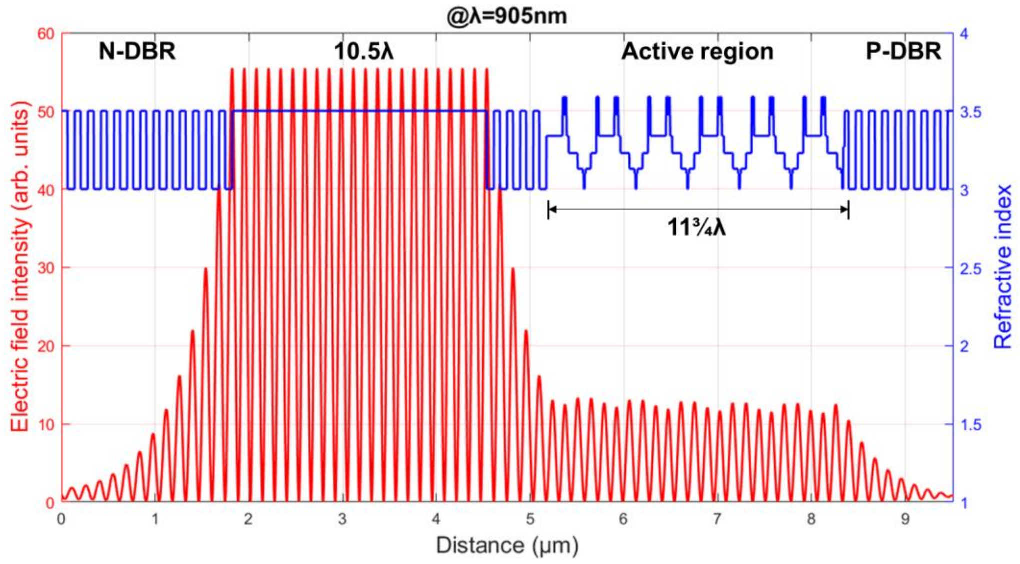

Figure S3(e). Replace the 11¾ λ spacer layer in Figure (d) with the active region having the same optical length (11¾ λ); use the 10.5 λ cavity as the light reservoir.

The 11¾ λ layer itself, where the active region is located, is anti-resonant, and remains so from Figure S3 c to d, and to e, determined by its optical length. Only together with the 10.5 λ length light reservoir and the middle antireflective DBR, they can be regarded as the resonant cavity, where the electric field intensity is the highest in the light reservoir. The combined optical length of the reservoir (11¾ λ), middle DBR (2.25 λ), and the active region (10.5 λ), is 24.5 λ, satisfying (1/2 + m/2) λ (m is a positive integer). Therefore, together they become one giant resonant cavity. Note that now the middle DBR is no longer part of the top DBR but becoming antireflective mirrors for the active region inside the giant cavity.

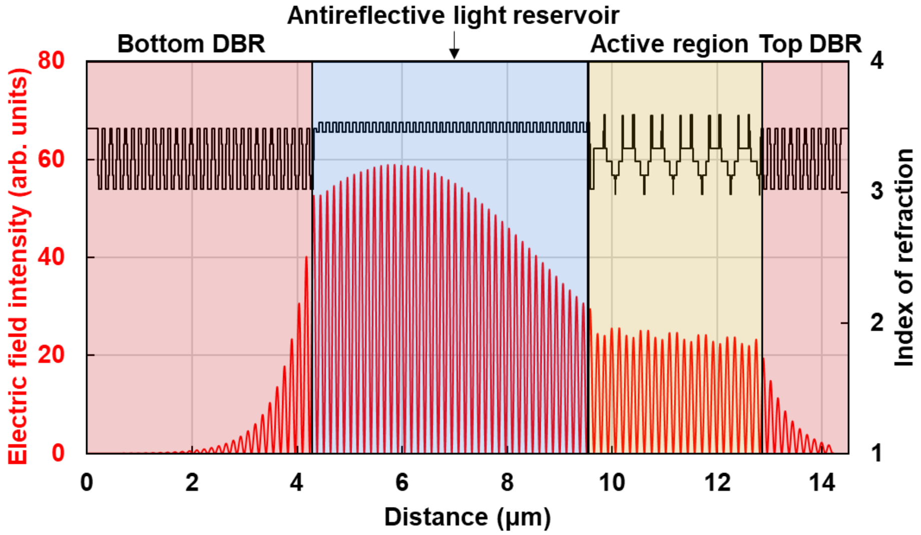

Figure S4 | An AR-VCSEL structure that integrates an antireflective mirror and a light reservoir forming an antireflective light reservoir.

A periodic structure can also be used throughout the light reservoir. The period can even be different from those for the top and bottom DBRs. As shown in Figure S4, a period slightly larger (or smaller) than half the lasing wavelength produces antireflection on the right part and reflection on the left. Collectively, these structures generate another positive (or negative) quarter wave shift, in addition to the initial quarter wave shift at the light reservoir-active region interface. The peak E-field position is intentionally shifted to the left by allocating more phase change on the left side so that the total stored E-field intensity can be even larger. The sum of the two quarter waves shifts, either cancelling each other out or adding up to a half wave shift, has zero effect on the phase of the bottom DBR, where the standing wave E-field intensity finally decreases.

C. Short pulse measurement details

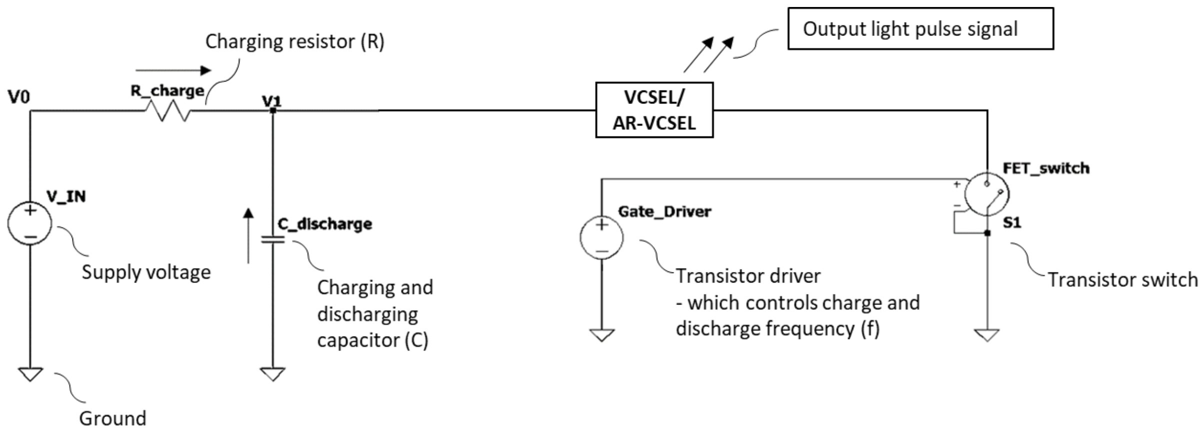

Figure S5 | A illustrative draft of the capacitor charge and discharge type short pulse generation circuit used for device measurement of the AR-VCSELs, our extended cavity VCSEL, the state-

of-the-art VCSEL and the state-of-the-art EEL. All measurements were conducted at room temperature.

D. Square AR-VCSEL array 13.5° (6J 100 µm) with 4 square emitters performance

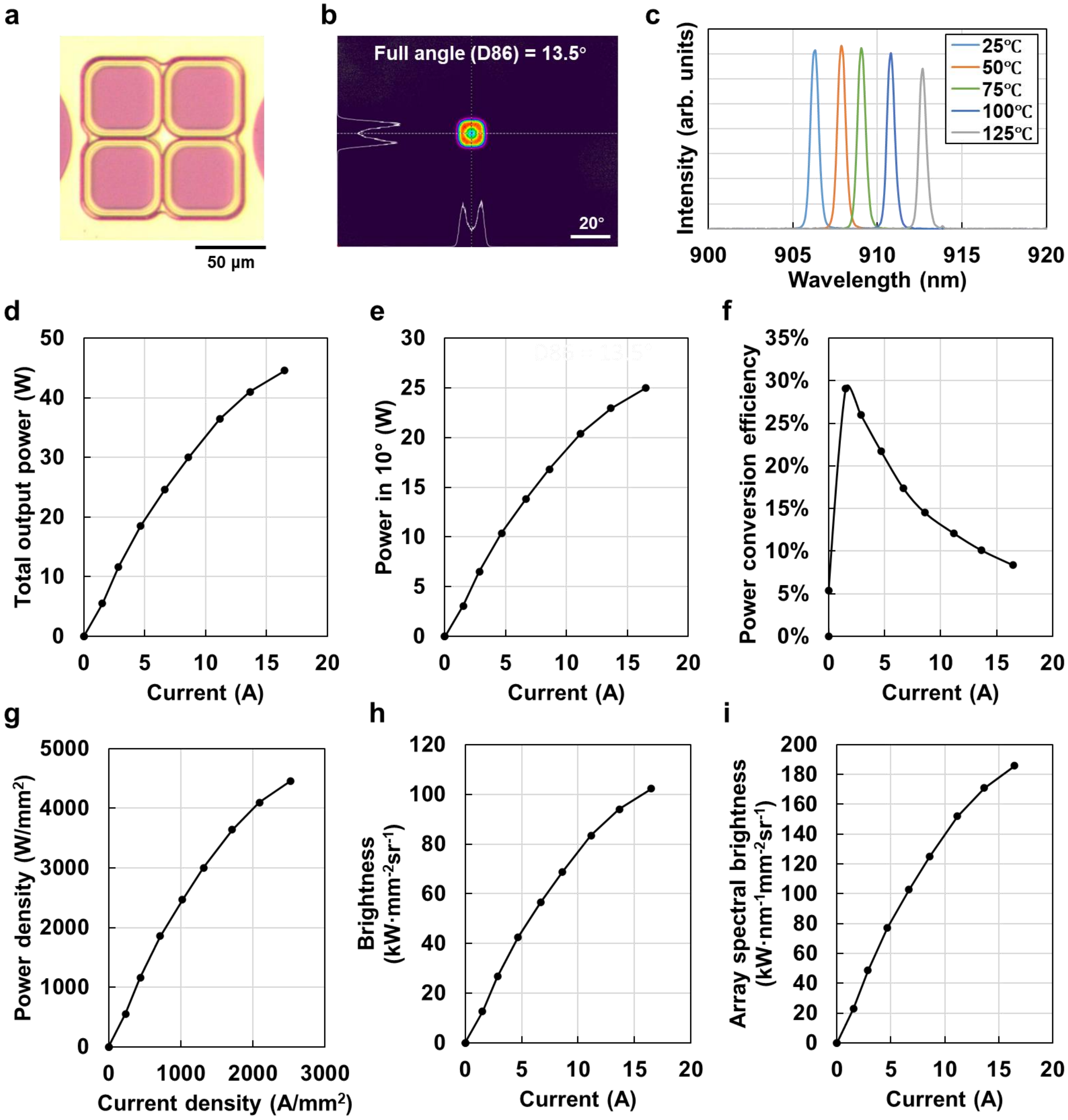

Figure S6 | Square AR-VCSEL array with 4 square emitters each having an optical aperture (OA) of 40 × 40 μm^2. (**a**) Device image. (**b**) Measured far field pattern at a current of 5.5 A. (**c**) Measured temperature dependent lasing spectrum at 5.5 A from 25 ℃ to 125 ℃. (**d-i**) Measured performance using the same method as the AR-VCSEL described in the main text.

E. 14J AR-VCSEL array 18.9° (14J 250 μm) performance

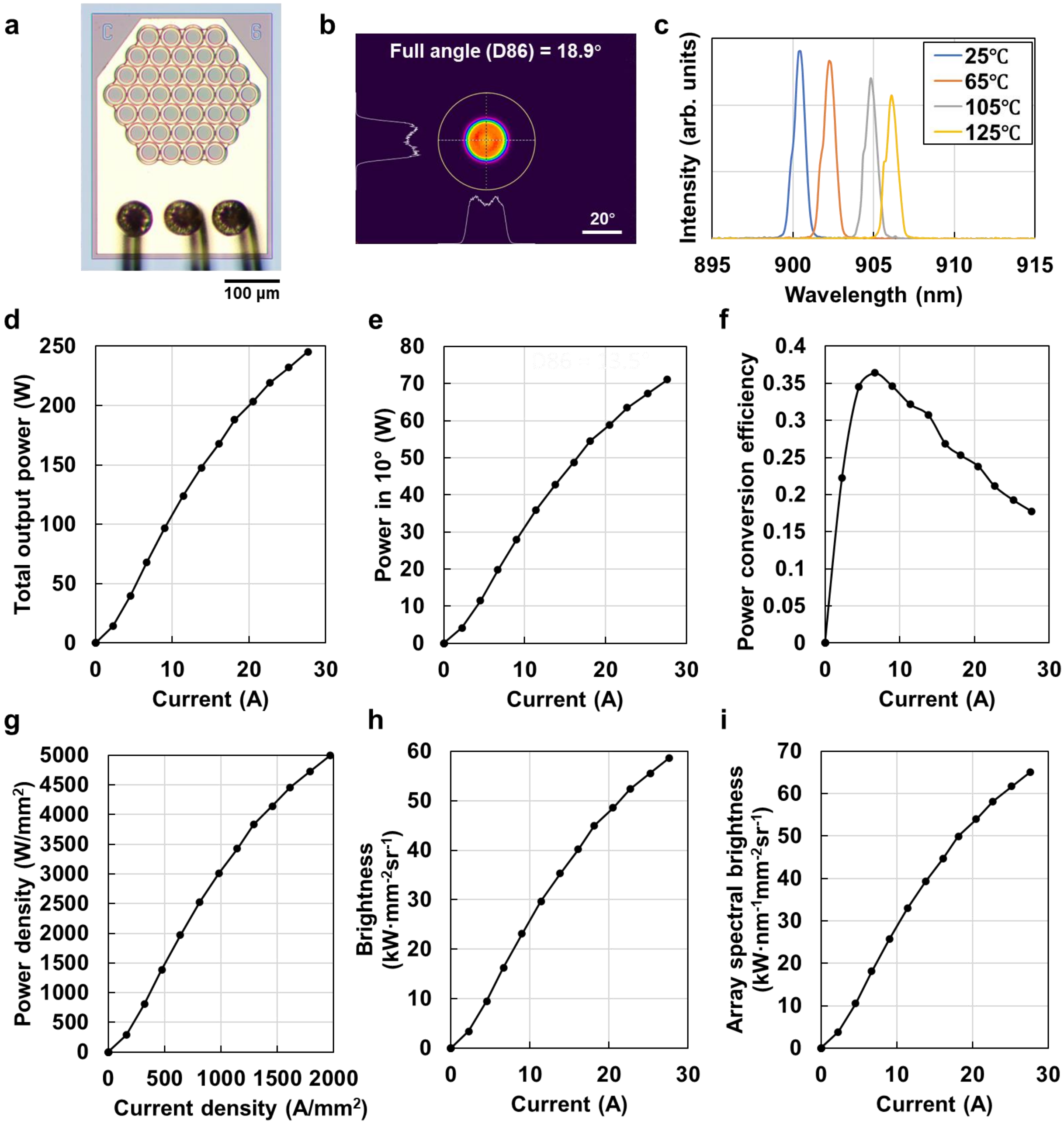

Figure S7 | 14J AR-VCSEL array with a hexagonally shaped emission area with a diameter of approximately 250 μm (each emitter has an OA of 22 μm in diameter). (**a**) Device image. (**b**) Measured far field pattern at a current of 10 A. (**c**) Measured temperature dependent lasing spectrum at 10 A from 25 ℃ to 125 ℃. (**d-i**) Measured performance using the same method as the AR-VCSEL described in the main text.

F. AR-VCSELs with various optical aperture (OA) diameters, ranging from 7 μm to 21 μm

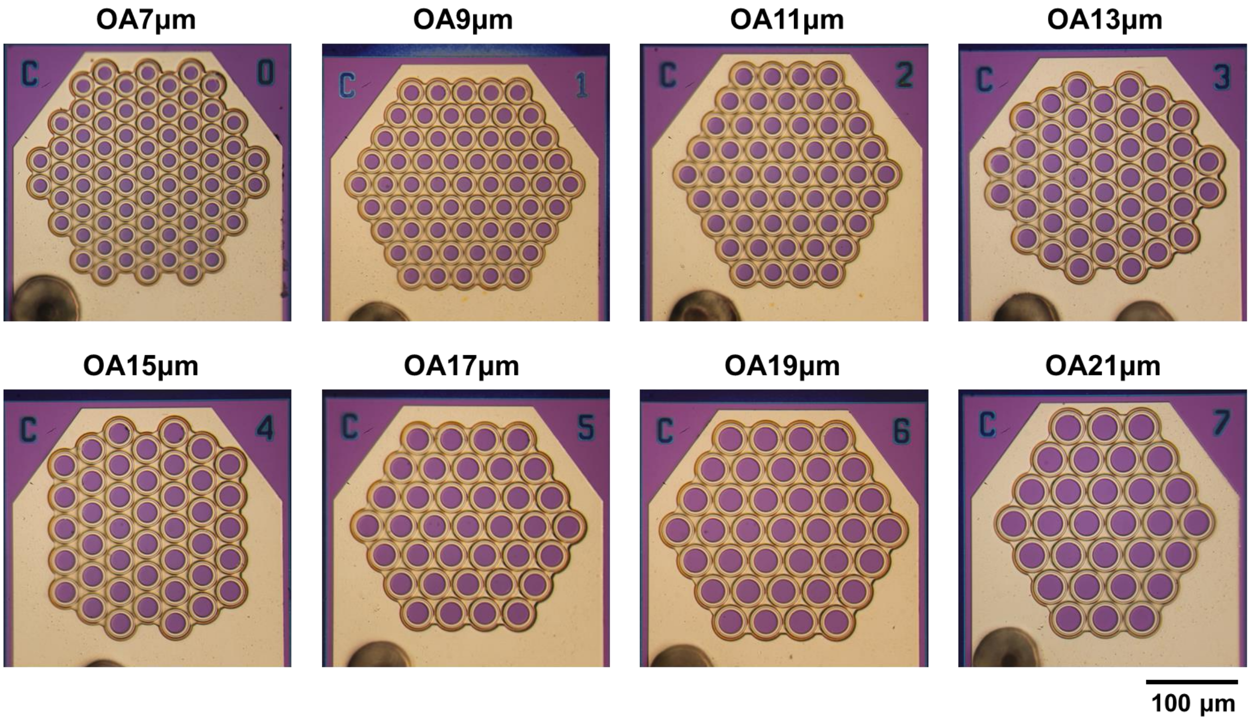

Figure S8 | Device images of AR-VCSEL arrays having optical aperture sizes ranging from 7 μm to 21 μm in diameter.

G. Single mode 6J VCSEL spectral measurement setup

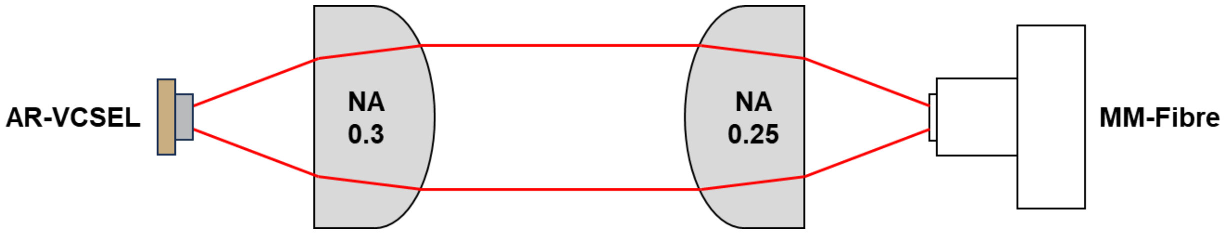

Figure S9 | Schematic of the free-space lens setup for single-emitter spectrum measurement.

H. Comparison between AR-VCSEL with conventional high power VCSEL and the-state-of-the-art PCSEL

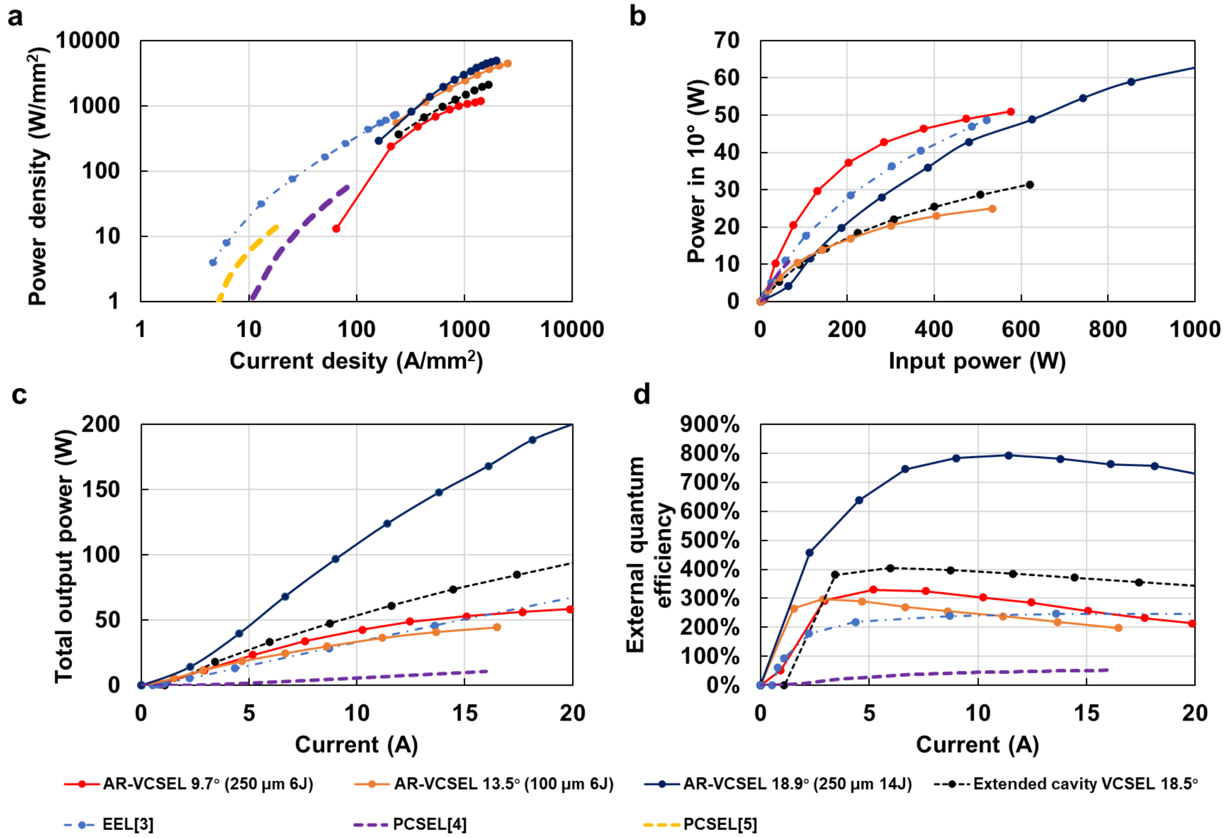

Figure S10 | Comparison between the AR-VCSELs and other types of semiconductor lasers for LiDAR applications[3–5]. **a-d**, Comparison of the measured power density versus the current density (**a**), the measured power within 10° FOV versus the input power (**b**), the measured total output power versus the current (**c**), and the measured external quantum efficiency versus the current (**d**) of the AR-VCSELs with other types of semiconductor lasers for LiDAR. The data of the state-of-the-art photonic-crystal surface-emitting laser (PCSEL) is extracted from Ref [4] (measured data, purple dashed line) and Ref [5] (theoretically calculated highest performance PCSEL, yellow dashed line) for LiDAR. The EEL's power (current) density is calculated by the power (current)

divided by the ridge area. All the surface-emitting lasers' power density is calculated by the power divided by the total emission area.

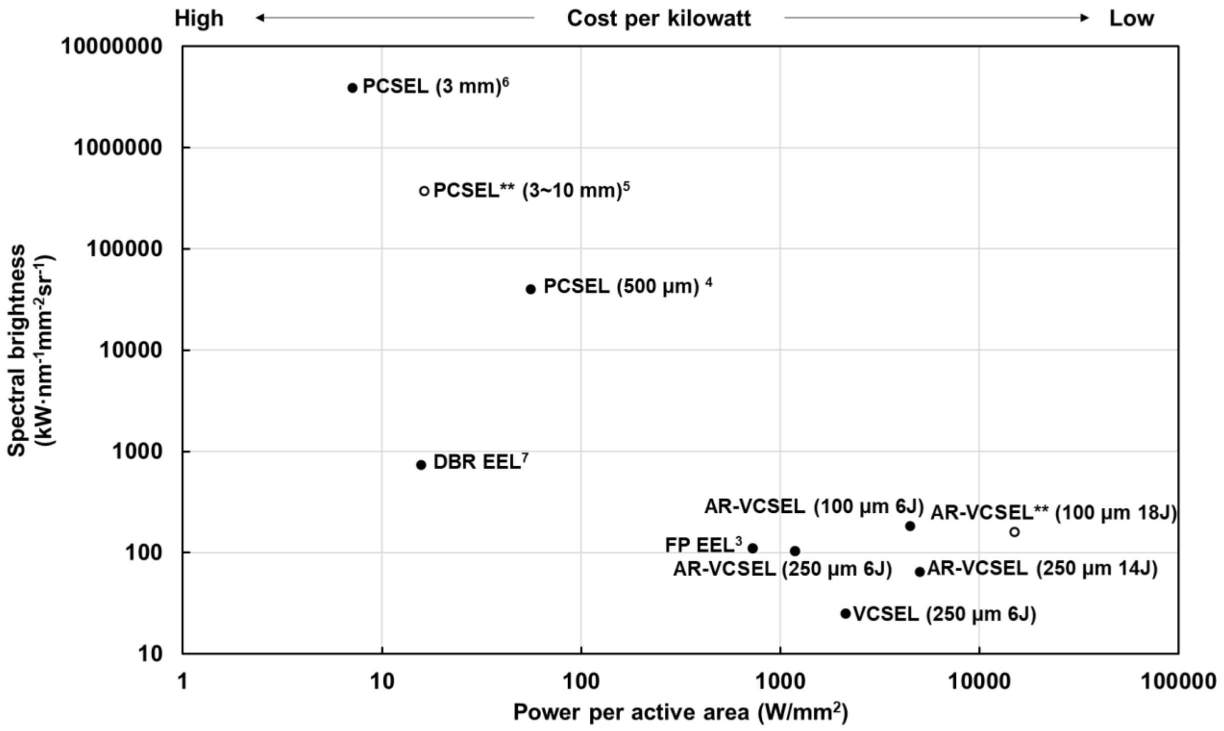

Figure S11 | Comparison of the spectral brightness between the AR-VCSELs and other types of semiconductor lasers for LiDAR applications[3–7].

There are two fundamental barriers that PCSELs need to overcome for a higher power density or an acceptable cost per kilowatt. The first barrier is the application of multijunction. The slope efficiency (Figure S10c) and the external quantum efficiency (Figure S10d) both reflect the importance of multijunction for power scalability. It is theoretically difficult to apply multijunction in PCSELs because effective coupling happens only when the photonic crystal (PC) layer is right next to a junction. Lacking a vertical standing wave, a junction's vertical light intensity profile has a large spreading of ~ 1 μm[8]. Like the EEL, a tunnel junction (TJ) must be placed in a minimum

of the optical field to avoid severe absorption loss. This spacing prevents the PC to couple with the light field of farther junctions unless each junction is paired with its own PC by extremely complicated fabrication and multiple times of regrowth. We believe integrating AR-VCSEL with PCSEL can possibly solve this problem, by placing the PC layer inside the light reservoir where the field antinodes are the strongest while aligning TJs with nodes where absorption is minimal. This is essentially a quasi-3D PC if we can match the vertical standing wavelength with the PC mode wavelength.

The second barrier comes from the rise of higher order modes of PC. Higher power density may break the single mode operation and significantly increase the divergence angle. An unstable divergence is a problem for LiDARs with fixed optics. Hirose et al.[9] in 2014 have shown that increasing the current density results in an increase of the beam divergence from 0.1° to 2°. Yoshida et al.[10] in 2019 did not show far field angle beyond 10 W. If we assume that a similar trend applies, e.g., a 2° angle at 40 W, then the brightness of PCSEL suddenly drops to a similar level as an AR-VCSEL can do. The unstable divergence regime of PCSEL must be avoided for scanning LiDAR with fixed optics. In contrast, AR-VCSEL's FF angle is very stable at higher current densities (Figure 3a in main text shows that the M^2 factor, which is proportional to the FF angle, is stable at different current injection levels).

References

1. Moser, P. Energy efficient oxide confined VCSELs for optical interconnects in data centers and supercomputers. (2015).
2. Zhang, C., ElAfandy, R. & Han, J. Distributed Bragg Reflectors for GaN-Based Vertical-Cavity Surface-Emitting Lasers. *Appl. Sci.* **9**, 1593 (2019).
3. Chip, SPL DS90A_3 | OSRAM Opto Semiconductors. https://www.osram.com/ecat/Chip%20SPL%20DS90A_3/com/en/class_pim_web_catalog_103489/prd_pim_device_2220026/.
4. Yoshida, M. *et al.* Photonic-crystal lasers with high-quality narrow-divergence symmetric beams and their application to LiDAR. *J. Phys. Photonics* **3**, 022006 (2021).
5. Inoue, T. *et al.* General recipe to realize photonic-crystal surface-emitting lasers with 100-W-to-1-kW single-mode operation. *Nat. Commun.* **13**, 3262 (2022).
6. Yoshida, M. *et al.* High-brightness scalable continuous-wave single-mode photonic-crystal laser. *Nature* **618**, 727–732 (2023).
7. Knigge, A. *et al.* Wavelength stabilized high pulse power laser bars for line-flash automotive LIDAR. in *High-Power Diode Laser Technology XVIII* vol. 11262 95–106 (SPIE, 2020).
8. Yang, Z.-X., Kuo, C.-Y. & Lin, G. Simulation of Photonic-Crystal Surface-Emitting Lasers with Air-Hole and Air-Pillar Structures. *Photonics* **8**, 189 (2021).
9. Hirose, K. *et al.* Watt-class high-power, high-beam-quality photonic-crystal lasers. *Nat. Photonics* **8**, 406–411 (2014).
10. Yoshida, M. *et al.* Double-lattice photonic-crystal resonators enabling high-brightness semiconductor lasers with symmetric narrow-divergence beams. *Nat. Mater.* **18**, 121–128 (2019).
